# Plasma Amino Acids in NAFLD Patients with Obesity Are Associated with Steatosis and Fibrosis: Results from the MAST4HEALTH Study

**DOI:** 10.3390/metabo13080959

**Published:** 2023-08-18

**Authors:** Athina I. Amanatidou, Eleni V. Mikropoulou, Charalampia Amerikanou, Maja Milanovic, Stefan Stojanoski, Mladen Bjelan, Lucia Cesarini, Jonica Campolo, Anastasia Thanopoulou, Rajarshi Banerjee, Mary Jo Kurth, Natasa Milic, Milica Medic-Stojanoska, Maria Giovanna Trivella, Sophie Visvikis-Siest, Amalia Gastaldelli, Maria Halabalaki, Andriana C. Kaliora, George V. Dedoussis, on behalf of the Mast4Health consortium

**Affiliations:** 1 Department of Nutrition and Dietetics, School of Health Science and Education, Harokopio University, 17671 Athens, Greece; camer@hua.gr (C.A.); dedousi@hua.gr (G.V.D.); 2 Division of Pharmacognosy and Natural Products Chemistry, Department of Pharmacy, National and Kapodistrian University of Athens, 15771 Athens, Greece; e.mikropoulou@pharm.uoa.gr (E.V.M.); mariahal@pharm.uoa.gr (M.H.); 3 Faculty of Medicine Novi Sad, University of Novi Sad, 21000 Novi Sad, Serbia; maja.milanovic@mf.uns.ac.rs (M.M.); stefan.stojanoski@mf.uns.ac.rs (S.S.); mladen.bjelan@gmail.com (M.B.); natasa.milic@mf.uns.ac.rs (N.M.); milica.medic-stojanoska@mf.uns.ac.rs (M.M.-S.); 4 Center for Diagnostic Imaging, Oncology Institute of Vojvodine, 21204 Sremska Kamenica, Serbia; 5 Division of Hepatology and Gastroenterology, ASST Grande Ospedale Metropolitano, 20162 Milan, Italy; lucia.cesarini@ospedaleniguarda.it; 6 Institute of Clinical Physiology, CNR, 56124 Milan, Italy; jonica.campolo@ospedaleniguarda.it; 7 Diabetes Center, 2nd Department of Internal Medicine, Medical School, National and Kapodistrian University of Athens, Hippokration General Hospital of Athens, 15772 Athens, Greece; athanop@med.uoa.gr; 8 Perspectum Ltd., Oxford OX4 2LL, UK; rajarshi.banerjee@perspectum-diagnostics.com; 9 Clinical Studies Group, Randox Laboratories Ltd., Crumlin BT29 4RN, UK; maryjo.kurth@randox.com; 10 Clinic for Endocrinology, Diabetes and Metabolic Diseases, Clinical Centre of Vojvodina, 21000 Novi Sad, Serbia; 11 Institute of Clinical Physiology National Research Council, 56124 Pisa, Italy; trivella@ifc.cnr.it (M.G.T.); amalia@ifc.cnr.it (A.G.); 12 ASST Grande Ospedale Metropolitano Niguarda, 20162 Milan, Italy; 13 INSERM UMR U1122, IGE-PCV, Faculté de Pharmacie, Université de Lorraine, 30 Rue Lionnois, 54000 Nancy, France; sophie.visvikis-siest@inserm.fr

**Keywords:** non-alcoholic fatty liver disease, magnetic resonance imaging, amino acids, metabolomics, inflammation

## Abstract

Non-alcoholic fatty liver disease (NAFLD) and non-alcoholic steatohepatitis (NASH) have been linked to changes in amino acid (AA) levels. The objective of the current study was to examine the relationship between MRI parameters that reflect inflammation and fibrosis and plasma AA concentrations in NAFLD patients. Plasma AA levels of 97 NAFLD patients from the MAST4HEALTH study were quantified with liquid chromatography. Medical, anthropometric and lifestyle characteristics were collected and biochemical parameters, as well as inflammatory and oxidative stress biomarkers, were measured. In total, subjects with a higher MRI-proton density fat fraction (MRI-PDFF) exhibited higher plasma AA levels compared to subjects with lower PDFF. The concentrations of BCAAs (*p*-Value: 0.03), AAAs (*p*-Value: 0.039), L-valine (*p*-Value: 0.029), L-tyrosine (*p*-Value: 0.039) and L-isoleucine (*p*-Value: 0.032) were found to be significantly higher in the higher PDFF group compared to lower group. Plasma AA levels varied according to MRI-PDFF. Significant associations were also demonstrated between AAs and MRI-PDFF and MRI-cT1, showing the potential utility of circulating AAs as diagnostic markers of NAFLD.

## 1. Introduction

Non-alcoholic fatty liver disease (NAFLD) is considered the leading cause of chronic liver disease in the world [1]. It represents a set of pathological conditions that range from simple hepatic steatosis (SS) or non-alcoholic fatty liver (NAFL) to non-alcoholic steatohepatitis (NASH) and cirrhosis [2]. Primary NAFLD is now acknowledged as the hepatic manifestation of metabolic syndrome (MetS) [3,4]. Processes that are involved in the onset of SS and its transition to NASH remain not fully explored.

NAFLD is linked to pathological disorders such as hypertension, insulin resistance (IR) and type II diabetes (T2D); obesity and increased central adiposity are also strongly associated with metabolic liver disease. High rates of obesity and T2D lead to an ever-increasing number of patients with NASH [5]. Despite efforts to develop new treatment strategies for NASH, no pharmaceutical medication has yet to receive approval. Due to the lack of identifiable symptoms, the disease is typically discovered later on, when attempts to treat it or reduce risk factors have failed [6]. Liver biopsy remains the gold standard method for disease diagnosis; however, it has significant drawbacks due to its invasive nature. LiverMultiScan™ (LMS, Perspectum Diagnostics, Oxford, UK) is a new multiparametric MRI software that has been successfully used in clinical trials to quantify fibrosis and inflammation [7] and to detect and stage liver disease [8,9].

The identification of non- or minimally invasive biomarkers that can track the progression of the disease or help to assess responses to therapeutic interventions is of upmost importance. In recent years, metabolomics has attracted a lot of scientific attention. Scientists are now able to identify hundreds of metabolites that are associated with several complex disorders thanks to the development of metabolomics. Given that urine or serum are the most commonly used samples for NAFLD testing, metabolomics is a valuable tool for assessing liver impairment. The variations in metabolite profiles of individuals with NAFLD have been the subject of numerous studies [10,11,12], with amino acids (AAs) being a particularly well-studied category that is altered at different stages of the disease [13]. Although it has recently been suggested that plasma AA levels could be used as potential markers of disease severity since they have been associated with IR and protein catabolism [14], few studies have addressed the relationship between AA levels and MRI parameters that reflect the disease.

The aim of the present study was to explore the relationship between MRI parameters that reflect inflammation and fibrosis and plasma AA concentrations in a NAFLD population. Such relationships broaden our knowledge and motivate further research on the use of plasma-free amino acid profiles as biomarkers for NAFLD diagnosis and prediction.

## 2. Materials and Methods

### 2.1. Study Design and Patients

This study used baseline data from a multicenter, randomized, double-blinded and placebo-controlled clinical trial (the MAST4HEALTH study [15], ClinicalTrials.gov (Identifier: NCT03135873) that explored the effect of Mastiha supplementation on liver inflammation and fibrosis in patients with NAFLD. In total, 97 participants were recruited (2017–2019) to three centers (the Department of Dietetics and Nutritional Science, Harokopio University, Athens, Greece (HUA); Consiglio Nazionale delle Ricerche Institute of Clinical Physiology, Milano section at Niguarda Hospital Italy (CNR); and Faculty of Medicine, University of Novi Sad, Serbia (UNS)) as previously described. Men and women aged 18–67 years with documented NAFLD/NASH based on the sensitive LiverMultiScan™ (LMS, Perspectum Diagnostics, Oxford, UK) [16]) and BMI > 30 kg/m^2^ were included in the research. Several exclusion criteria were applied and are extensively elsewhere described [15], such as decompensated diabetes mellitus, hepatotoxic medication, alcohol abuse [>20 g day^−1^ (women), >30 g day^−1^ (men), EASL Guidelines], pregnancy, etc. All centers obtained ethics committee approvals (HUA (Bioethics Committee 49/29 October 2015), CNR (Ethical Clearance by Commissione per l’Etica e l’Integrità nella Ricerca, February 2016) and Niguarda Hospital Ethics Committee 230-052017 (Comitato Etico Milano Area 3-11.05.2017), UNS (Faculty of Medicine Novi Sad, The Human Research Ethics Commission No. 01-39/58/1-27.06.2016)), and the trial was carried out in accordance with the rules of the Declaration of Helsinki and the Data Protection Act of 1998. Before being included in the study, all participants provided written informed consent.

### 2.2. Medical, Anthropometric and Lifestyle Assessments 

Detailed questionnaires on medical history and lifestyle were obtained. To estimate T2D risk, the Finnish diabetic risk score (FINDRISK) questionnaire was used. The questions pertain to age, BMI, waist circumference (WC), physical activity, vegetable and fruit consumption, hypertension, and personal and family history of hyperglycemia [17]. Physical activity level (PAL) was measured using the international physical activity questionnaire (IPAQ) [18], and metabolic equivalent task minutes per week (MET-min/week) was calculated using the IPAQ scoring system. Interviewers classified participants as smokers or nonsmokers based on their smoking status [19]. Body weight, height and waist circumference were measured, and body mass index (BMI) was computed by dividing weight (kg) by height (m)^2^. Waist and hip circumference were measured and waist to hip ratio (WHR) was computed. Nutritionist Pro™ (Axxya Systems) was used to assess the dietary intake based on 24 h recalls (three randomly selected days).

### 2.3. MRI Parameters

MRI parameters [Magnetic Resonance Imaging Iron-corrected T1 (cT1), proton density fat fraction (PDFF) and liver inflammation fibrosis score (LIF)] were derived from the use of LiverMultiscan software on the MRIs of the participants [16].

### 2.4. Blood Collection

- Biochemical parameters

Blood collection (25 mL) was performed during the baseline visit after an overnight fast and serum and plasma isolation was conducted after centrifugation (3000 rpm, 10 min) [15]. The serum was used for the measurement of liver enzymes (glutamyltransferase (gamma-GT), aspartate transaminase (AST), and alanine transaminase (ALT)), lipids (total cholesterol, high-density lipoprotein (HDL), low-density lipoprotein (LDL), and triglycerides (TG)), glucose and insulin [15]. Also, HOMA-IR was calculated as follows: fasting glucose (mg/dL) × (fasting insulin)/405 and 75 g of the 2 h oral glucose tolerance test (OGTT) was performed.

The plasma was stored at −80 °C until further use for metabolomics analysis.

- Inflammation and oxidative stress biomarkers

Total antioxidant status (TAS) (mmol/L) was determined in serum by Randox TAS kits (Randox Laboratories Ltd., Crumlin, UK) at Randox Clinical Laboratory Services (Antrim, UK). Superoxide Dismutase (SOD) activity was measured with the RANSOD kit (Randox Laboratories Ltd., Crumlin, UK) in an erythrocyte pellet using a Randox RX Series Analyser (Randox Laboratories Ltd., Crumlin, UK). IL-1α, IL-1β, IL-2, IL-4, IL-6, IL-8, IL-10, MCP-1, TNF-α, INF-γ, EGF and VEGF-A were quantified in serum with the Randox high sensitivity cytokine I multiplex array (Randox Laboratories Ltd., Crumlin, UK), in an Evidence Investigator analyser (Randox Laboratories Ltd., Crumlin, UK).

### 2.5. Plasma Amino Acid Profiles

- Sample preparation and labeling with the aTRAQ^®^ reagents

Sample preparation was based on amino acid derivatization using the aTRAQ^®^ reagents (AB Sciex, MA, USA) as previously described [20]. In brief, 10 μL of 10% sulfosalicylic acid containing 400 pmol/μL of norleucine were added to 40 μL of plasma for protein precipitation. A 10 μL volume of the supernatant was mixed with 40 μL of labeling buffer containing 20 pmol/μL of norvaline. A 10 μL volume of the supernatant was mixed with 5 μL of 121 aTRAQ^®^ labeling reagent. The samples were incubated for 30 min at room temperature and finally 5 μL of hydroxylamine was added. The samples were dried using an Eppendorf vacufuge concentrator and reconstituted to 32 μL of 113 aTRAQ^®^ internal standard diluted with 0.2% formic acid in water at a ratio of 1:1.

- Separation and detection

Liquid chromatography analysis was performed on an Acquity UPLC^®^ system (Waters, MA, USA) equipped with a binary solvent pump. For detection, a TripleTOF^®^ 5600+ mass spectrometer was employed (AB Sciex), equipped with a DuoSpray™ ion source operated in the positive ESI mode. Injection volume was set to 2 μL and separation was carried out on an Amino Acid Analyzer C18 Reversed Phase column (5 µm, 4.6 mm × 150 mm, AB Sciex) using a gradient composed of water (Millipore Direct-Q 3 UV purification system, Millipore Sigma, MA, USA) and methanol (MS grade, J.T. Baker, NJ, USA) both containing 0.1% formic and 0.01% heptafluorobutyric acid. The column temperature was set to 50 °C and the flow rate was 0.8 mL/min. Analyte determination was based on a variable-window SWATH acquisition method. For the ESI source, the temperature was set to 600 °C and the ion spray voltage was 4500 V. Source gas and exhaust gas were both set to 60 psi and curtain gas was set to 30 psi. Data acquisition was performed using the Analyst^®^ 1.7.1 software, while processing was achieved using the Sciex OS software platform.

### 2.6. Statistical Analysis

The R programming language (R Foundation, Vienna, Austria) was used for data management and analysis. The variables are presented as mean ± standard deviation (SD). The Shapiro–Wilk test was performed to evaluate the variable distributions. The differences in variables across groups were assessed using independent samples *t*-test for normally distributed or Mann–Whitney U test for non-normally distributed variables, and x-squared test for categorical variables. To analyze plasma AA level differences across PDFF and cT1 tertiles (PDFF: 1st tertile: 7.895, 3rd tertile: 22.080; cT1: 1st tertile: 828.9, 3rd tertile: 916.6), the analysis of variance (ANOVA) using Tukey’s post hoc test was applied for all normally distributed variables and the Kruskal–Wallis test using Dunn’s post hoc test was applied for all variables that did not follow a normal distribution. Pairwise comparisons were performed using the Benjamini–Hochberg correction. Using the Bonferroni correction, the resulting *p*-Values for plasma AA level differences across PDFF and cT1 tertiles were corrected. Pearson’s correlation coefficient for parametric variables or Spearman’s rank correlation for non-parametric variables were estimated to determine the correlation between AA concentrations and other tested parameters. In order to address the issue of multiple comparisons in correlation analysis, the Holm–Bonferroni method was applied. Linear regression models were created to detect statistically significant associations of AAs with PDFF and cT1. Due to the skewness of the distribution, the PDFF and cT1 were log transformed. *p*-Values were corrected for multiple testing using the Bonferroni correction. Five adjustment sets were considered: Model 1—crude; Model 2—adjusted for age + sex; Model 3—adjusted for age + sex + BMI; and Model 4—adjusted for age + sex + BMI + PAL + smoking + center of the study; Model 5—adjusted for age + sex + BMI + PAL + smoking + center of the study + nutrient intake of the specific AA. In all tests, a *p*-Value < 0.05 was deemed significant.

## 3. Results

### 3.1. General Characteristics of Study Participants

The current study included 97 individuals, with a total of 69 males and 28 females, and a mean age of 49.04 ± 9.16. Table 1 displays the descriptive characteristics of the population. ALT was found to be significantly higher in males than in females (*p*-Value: 0.001). Moreover, females had significantly higher AST/ALT ratios, total cholesterol, HDL, LDL, HGB and TAS than males (AST/ALT ratio, *p*-Value: 0.004; total cholesterol, *p*-Value: 0.010; HDL, *p*-Value: 0.022; LDL, *p*-Value: 0.028; HGB, *p*-Value: 1.37 × 10^−9^; TAS, *p*-Value: 4.94 ×10^−4^).

### 3.2. AA Plasma Levels across PDFF and cT1 Categories

PDFF and cT1 were grouped into tertiles (low, medium and high) (Table 2). The plasma AA levels (Table 2) of 5 of the 39 AAs differed significantly within PDFF tertiles after correction for multiple testing. The concentrations of BCAAs (*p*-Value: 0.03), AAAs (*p*-Value: 0.039), L-valine (*p*-Value: 0.029), L-tyrosine (*p*-Value: 0.039) and L-isoleucine (*p*-Value: 0.032) were found to be significantly higher in the “high” and “medium” PDFF groups compared to the “low” group.

### 3.3. Correlations of AAs with MRI-PDFF and Other Disease Parameters

In the correlation analysis, various statistically positive correlations were observed (Table S1). The AAAs (r: 0.44), L-tyrosine (r: 0.44) and L-isoleucine (r: 0.42) exhibited positive correlations with PDFF. In addition, ethanolamine (HGB, r: 0.44|TAS, r: 0.43) was positively correlated with hemoglobin levels (HGB) and total antioxidant status (TAS), and L-ornithine (r: 0.44) with TAS. The essential AAs (insulin, r: 0.42, HOMA-IR, r: 0.47), BCAAs (insulin, r: 0.48, HOMA-IR, r: 0.52), AAAs (insulin, r: 0.46, HOMA-IR, r: 0.47), L-proline (insulin, r: 0.44, HOMA-IR, r: 0.43), L-valine (insulin, r: 0.44, HOMA-IR, r: 0.48), L-isoleucine (insulin, r: 0.42, HOMA-IR, r: 0.48), L-leucine (insulin, r: 0.45, HOMA-IR, r: 0.47) and L-phenylalanine (insulin, r: 0.47, HOMA-IR, r: 0.44) were positively correlated with insulin and HOMA-IR. Also, L-methionine (r: 0.38) was positively correlated with insulin and L-tyrosine (r: 0.43) with HOMA-IR. ALT was found to be positively correlated with AAAs (r:0.43), L-phenylalanine (r: 0.44) and L-tryptophan (r: 0.42).

### 3.4. Associations of AAs with MRI-PDFF and MRI-cT1

Linear regression models were created to explore the associations of AAs with MRI-PDFF and MRI-cT1 (Table 3, Tables S2 and S3). In Model 1, the essential AAs (exp(beta): 1.002, *p*-Value: 0.037), GSG index (exp(beta): 1.032, *p*-Value: 0.019), BCAAs (exp(beta): 1.003, *p*-Value: 0.014), AAAs (exp(beta): 1.013, *p*-Value: 0.001), L-glutamic acid (exp(beta): 1.015, *p*-Value: 0.006), L-valine (exp(beta): 1.005, *p*-Value: 0.005), L-tyrosine (exp(beta): 1.018, *p*-Value: 0.003) and L-phenylalanine (exp(beta): 1.029, *p*-Value: 0.014) were associated with increased values of log-PDFF (Table 3). The associations of AAAs (Model 2: exp(beta): 1.012, *p*-Value: 0.008|Model 3: exp(beta): 1.013, *p*-Value: 0.006), L-glutamic acid (Model 2: exp(beta): 1.014, *p*-Value: 0.019|Model 3: exp(beta): 1.014, *p*-Value: 0.025), L-valine (Model 2: exp(beta): 1.005, *p*-Value: 0.018|Model 3: exp(beta): 1.005 *p*-Value: 0.024) and L-tyrosine (Model 2: exp(beta): 1.016, *p*-Value: 0.014|Model 3: exp(beta): 1.017, *p*-Value: 0.010) with log-PDFF remained statistically significant in Models 2 and 3. In Models 4 and 5, BCAAs (Model 4: exp(beta): 1.003, *p*-Value: 0.037|Model 5: exp(beta): 1.003, *p*-Value: 0.040), AAAs (Model 4: exp(beta): 1.012, *p*-Value: 0.043 | Model 5: exp(beta): 1.012, *p*-Value: 0.036), L-glutamic acid (Model 4: exp(beta): 1.015, *p*-Value: 0.026|Model 5: exp(beta): 1.016, *p*-Value: 0.031) and L-valine (Model 4: exp(beta): 1.005, *p*-Value: 0.009|Model 5: exp(beta): 1.005, *p*-Value: 0.010) were found to be associated with increased log-PDFF values (Table 3).

The L-glutamic acid was associated with higher log-cT1 values across all models (Model 1: exp(beta): 1.002, *p*-Value: 0.018|Model 2: exp(beta): 1.002, *p*-Value: 0.014|Model 3: exp(beta): 1.002, *p*-Value: 0.043|Model 4: exp(beta): 1.002, *p*-Value: 0.043|Model 5: exp(beta): 1.002, *p*-Value: 0.031). In Models 2–5, L-threonine was linked to higher log-cT1 values (Model 2: exp(beta): 1.001, *p*-Value: 0.041|Model 3: exp(beta): 1.001, *p*-Value: 0.008|Model 4: exp(beta): 1.001, *p*-Value: 0.001|Model 5: exp(beta): 1.001, *p*-Value: 0.001). AAAs were also associated with greater values of log-cT1 in Model 3 (exp(beta): 1.001, *p*-Value: 0.038) (Table 3).

## 4. Discussion

Different levels of several plasma AAs across the PDFF and cT1 categories were identified herein. Additionally, significant correlations were observed between several plasma AA levels and MRI-PDFF and other disease parameters (HGB, TAS, insulin, HOMA-IR and ALT). Using linear regression models, statistically significant associations were found between AA concentrations and MRI-PDFF and MRI-cT1.

Our findings showed elevated plasma levels of the GSG index, BCAAs, L-valine, L-tyrosine and L-isoleucine in the “high” and “medium” PDFF groups compared to the “low” PDFF group. Interestingly, several statistically significant associations between plasma AAs and log-PDFF and log-cT1 were detected. More specifically, essential AAs, GSG index, BCAAs, AAAs, L-glutamic acid, L-valine, L-tyrosine and L-phenylalanine were associated with increased values of log-PDFF. Furthermore, L-glutamic acid, L-threonine and AAAs were also associated with greater log-cT1 values. Our results support the findings of other research studies who showed the associations of the above AAs with more advanced stages of this disease.

Previous studies have shown positive associations between plasma valine, isoleucine and leucine, with intrahepatic lipid content [21]. Higher plasma BCAA levels were determined and correlated with MRI-PDFF even in children with NAFLD [22].

Recent research suggested that the GSG index, which incorporates three amino acids essential for the production of glutathione, may be a promising biomarker of NAFLD [14,23]. According to a study by Ajaz et al. [24], its component glutamate was found to be significantly higher in NASH patients with severe fibrosis, whereas glycine and serine had a negative association with the degree of steatosis [25]. Alanine, a nonessential AA, and valine and methionine, essential AAs, are involved in the development of NASH [26]. The liver–alpha cell axis is thought to be significantly regulated by alanine [27]. Lysine was present in higher concentrations in NAFLD patients with grade 2 hepatocellular ballooning than in healthy controls, and NAFLD patients overall [14]. Our findings are also consistent with patients who had higher BCAA values along with more severe liver impairment [14,28,29], which is also reflected in our results. The study of Lake et al. [30] showed that serum levels of the BCAAs leucine, isoleucine and valine considerably increase as steatosis developed into NASH. This rise is linked to hepatic fat accumulation in early stages of NAFLD. 

As observed in several studies, AAA levels are higher in NASH and SS patients compared to controls [12,31]. Interestingly, patients with NASH have higher serum levels of the AAAs tyrosine, phenylalanine and tryptophan [32]. Of note, phenylalanine was found to be higher in NAFLD, NASH and obesity; tyrosine was associated with IR and the NASH fibrotic stage; tryptophan was found to be higher in NASH compared to SS or controls and not in SS compared to controls, indicating its potential contribution to liver fibrosis or inflammation [12,14,32,33,34,35]. Previously, glutamic acid concentration was found to be altered in NAFLD, probably due to its involvement in glutathione formation and possibly connected to the severity of NAFLD [14]. It was recently found that greater threonine intake was inversely associated with NAFLD risk in elderly Chinese people [36]. In contrast, the current study found that higher plasma threonine concentrations were associated with higher MRI-cT1. The conflicting results could be attributed to genetic and environmental differences between Asian and Western countries, and they need be validated in future research.

While the results of this study are intriguing, it has some limitations, including the small number of participants, the lack of biopsies for the staging of the disease and the inclusion of a multiethnic population. However, the aforesaid limitations are mitigated by the use of LiverMultiscan, a sensitive software with satisfactory diagnostic accuracy, the adjustment of several confounders, such as the center of the study, and the application of a highly sensitive LC method.

In conclusion, different plasma AA levels were observed according to different MRI clinical variables. Also, we reported some interesting associations between MRI-PDFF and MRI-cT1 that reflect disease activity and plasma AAs for the first time. The above findings suggest a potential utility of AAs as predictive markers of disease progression. Since the limited sample size makes these results preliminary, additional research with extensive predictive models and validation with other databases are needed in the future to support our conclusions.

## Figures and Tables

**Table 1:** Select baseline characteristics of the population.

Variables	Ν	All (Mean (SD))	Females (N: 28) (Mean (SD))	Males (N:69) (Mean (SD))	*p*-Value
**Age (years)**	97	49.04 (9.16)	49.61 (7.67)	48.81 (9.74)	0.676
**Smoking (Yes|No)**	96	Yes: 21, No: 75	Yes: 7, No: 21	Yes: 14, No:54	0.839
**BMI (kg/m^2^)**	97	34.43 (4.43)	35.39 (5.19)	34.04 (4.06)	0.228
**PAL (total MET- min/week)**	91	3622.17 (5128.17)	3733.37 (5326.04)	3575.26 (5084.72)	0.452
**cT1 (ms)**	94	877.94 (79.82)	874.27 (65.96)	879.5 (85.43)	0.82
**PDFF (%)**	95	16.59 (11.98)	12.89 (8.14)	18.06 (12.96)	0.058
LIF *	94	2.26 (0.63)	2.25 (0.59)	2.27 (0.65)	0.902
**AST (IU/L)**	94	25.39 (11.13)	22.59 (8.15)	26.52 (12)	0.093
**ALT (IU/L)**	94	38 (20.44)	28.93 (14.97)	41.66 (21.29)	**0.001**
**AST/ALT ratio**	94	0.74 (0.24)	0.88 (0.32)	0.68 (0.17)	**0.004**
**γ-gt (U/L)**	96	55. 37 (60.48)	62.04 (79.03)	52.77 (51.95)	0.28
**Total cholesterol (mg/dL)**	97	196.39 (37.48)	209.01 (33.5)	191.27 (38.03)	**0.010**
**HDL (mg/dL)**	97	44.51 (10.39)	48 (11.17)	43.1 (9.8)	**0.022**
**LDL (mg/dL)**	96	122.23 (34.92)	130.95 (30.35)	118.64 (36.23)	**0.028**
**Triglycerides (mg/dL)**	97	148.57 (65.33)	150.54 (76.47)	147.77 (60.83)	0.793
**Glucose (mg/dL)**	92	102.59 (15.65)	98.84 (10.35)	104.15 (17.21)	0.343
**120 min-OGTT Glucose (mg/dL)**	86	131.99 (47.58)	131.35 (38.08)	132.26 (51.45)	0.665
**HOMA-IR**	89	4.89 (2.61)	4.23 (2.43)	5.19 (2.65)	0.109
**Insulin (μU/mL)**	93	19 (9.83)	16.83 (10.18)	19.93 (9.6)	0.096
HGB (g/mL) *	96	0.15 (0.01)	0.135 (0.009)	0.151 (0.011)	**1.37 × 10^−9^**
**TAS (mmol/L)**	96	1.91 (0.21)	1.791 (0.177)	1.951 (0.202)	**4.94 × 10^−4^**

Note: * normally distributed variable. *p*-Value for comparison between females and males was obtained using *t*-test for normally distributed variables or Mann–Whitney U test for non-normally distributed variables, and the chi-square test for categorical variables. Bold *p*-Values show statistical significance. PAL: physical activity level; cT1: included iron-corrected; PDFF: proton density fat fraction; LIF: liver inflammation fibrosis score; AST: aspartate transaminase; ALT: alanine transaminase; AST/ALT ratio: AST to ALT ratio; γ-GT: γ-glutamyltransferase; HDL: high-density lipoprotein; LDL: low-density lipoprotein; HOMA-IR: homeostatic model assessment of insulin resistance; HGB: hemoglobin level: (g/mL); TAS: total antioxidant status, mean (mmol/L).

**Table 2:** Plasma AA levels in PDFF and cT1 categories.

Amino Acids (AAs) μmoles/L	PDFF (%)			*p*-Value	Corrected *p*-Value ^c^	cT1 (ms)			*p*-Value	Corrected *p*-Value ^c^
	Low (N: 24) Mean (SD)	Medium (N: 47) Mean (SD)	High (N: 24) Mean (SD)			Low (N: 24) Mean (SD)	Medium (N: 46) Mean (SD)	High (N: 24) Mean (SD)		
**Essential AAs**	1010.507 (121.086) # *	1117.744 (172.683) #	1147.612 (126.467) *	0.002	0.078	1044.921 (110.025) *	1097.495 (188.893)	1144.300 (121.150) *	0.035	1.000
**Nonessential AAs**	1603.642 (173.144)	1630.705 (196.743)	1703.354 (307.453)	0.442	1.000	1572.581 (167.286)	1629.763 (180.126)	1698.318 (335.970)	0.236	1.000
**GSG Index**	13.940 (7.202) *	16.371 (6.675)	20.605 (9.649) *	0.038	1.000	15.863 (6.368)	15.876 (7.683)	19.698 (9.553)	0.234	1.000
**BCAAs**	441.535 (74.955) # *	521.277 (109.371) #	527.588 (75.433) *	7.6 × 10^−4^	**0.03**	476.257 (71.391)	504.378 (122.071)	522.531 (71.487)	0.133	1.000
**AAAs**	127.859 (15.436) # *	141.767 (21.725) #	150.859 (24.663) *	0.001	**0.039**	130.773 (17.302)	140.170 (22.655)	148.765 (24.896)	0.052	1.000
L-Alanine *	317.202 (56.578)	337.971 (58.528)	350.397 (56.300)	0.133	1.000	309.325 (52.834)	343.036 (58.583)	337.085 (55.198)	0.059	1.000
Beta-Alanine *	7.026 (1.746)	7.847 (1.822)	7.984 (1.959)	0.136	1.000	7.238 (1.719)	7.920 (1.817)	7.786 (1.871)	0.32	1.000
**Sarcosine**	3.579 (1.368)	3.922 (1.454)	3.702 (1.457)	0.291	1.000	3.600 (1.244)	3.812 (1.490)	3.924 (1.531)	0.65	1.000
**Cystine**	40.717 (14.985)	39.377 (18.192)	39.065 (16.011)	0.959	1.000	40.023 (14.163)	37.813 (17.141)	40.061 (18.420)	0.739	1.000
**L-Serine**	96.910 (20.859)	100.830 (48.638)	123.573 (164.319)	0.703	1.000	92.250 (16.841)	96.555 (18.828)	135.814 (174.332)	0.587	1.000
**O-Phosphoethanolamine**	1.942 (2.186)	1.443 (1.721)	1.549 (1.849)	0.577	1.000	1.145 (1.217)	1.760 (1.983)	1.672 (2.120)	0.555	1.000
**Taurine**	53.064 (20.481)	61.665 (22.178) **	48.514 (15.845) **	0.024	0.936	53.257 (15.357)	55.314 (22.141)	58.918 (24.626)	0.68	1.000
**L-Asparagine**	54.496 (11.271)	53.350 (7.353)	55.100 (6.664)	0.52	1.000	51.039 (9.143)	54.766 (8.654)	54.270 (7.440)	0.175	1.000
**Hydroxy-L-Proline**	11.304 (5.339)	13.959 (9.471)	13.035 (4.640)	0.216	1.000	11.515 (5.288)	14.474 (9.701)	11.349 (3.616)	0.359	1.000
**Glycine**	226.806 (64.259)	198.042 (43.774)	204.405 (95.119)	0.049	1.000	200.584 (34.363)	206.239 (56.277)	213.808 (100.801)	0.649	1.000
L-Glutamine *	567.882 (57.524)	584.667 (73.382)	581.345 (62.948)	0.604	1.000	576.075 (74.131)	576.024 (71.608)	577.389 (51.543)	0.996	1.000
Ethanolamine *	6.941 (0.999)	7.175 (1.157)	7.136 (0.904)	0.672	1.000	7.089 (0.964)	7.082 (1.121)	7.161 (1.113)	0.956	1.000
**L-Aspartic Acid**	2.774 (1.226)	3.516 (2.018)	3.142 (0.938)	0.0841	1.000	2.873 (1.376)	3.305 (2.004)	3.406 (1.040)	0.073	1.000
**L-Citruline**	34.583 (6.578)	33.641 (8.581)	33.246 (7.836)	0.734	1.000	33.913 (6.809)	32.740 (8.673)	33.851 (6.616)	0.542	1.000
**L-Threonine**	119.821 (21.051)	118.390 (25.142)	134.106 (38.418)	0.136	1.000	113.123 (22.263) *	119.272 (22.610)	137.588 (39.616) *	0.037	1.000
**L-Glutamic Acid**	40.962 (17.351) *	46.498 (15.045)	58.379 (22.542) *	0.024	0.936	44.725 (15.954)	45.112 (18.403)	57.037 (19.981)	0.05	1.000
L-Histidine *	80.343 (7.429)	83.616 (12.239)	81.368 (12.152)	0.466	1.000	80.325 (10.822)	82.975 (9.536)	82.066 (14.450)	0.649	1.000
**1-Me-L-Histdine**	7.062 (7.218)	9.403 (9.220)	9.013 (6.706)	0.682	1.000	7.591 (7.616)	9.315 (9.383)	8.858 (6.398)	0.538	1.000
**3-Me-L-Histdine**	3.588 (1.080)	4.212 (1.217)	4.146 (1.264)	0.0619	1.000	4.085 (1.317)	4.061 (1.257)	4.037 (1.116)	0.969	1.000
**Gamma-Amino-Butyric Acid (GABA)**	0.253 (0.198)	0.261 (0.197)	0.235 (0.198)	0.686	1.000	0.240 (0.169)	0.268 (0.188)	0.234 (0.205)	0.42	1.000
**D,L-Beta-Aminoisobutyric Acid**	1.329 (0.951)	1.534 (3.636)	0.931 (0.474)	0.133	1.000	2.190 (5.040)	1.004 (0.525)	1.003 (0.500)	0.434	1.000
**D,L-Alpha-Amino-n-Butyric Acid**	18.008 (4.930)	21.551 (8.995)	19.206 (6.469)	0.249	1.000	20.012 (6.643)	19.911 (8.635)	20.533 (7.090)	0.868	1.000
**L-Alpha-Aminoadipic Acid**	1.133 (0.665)	2.316 (6.846)	1.406 (0.343)	0.173	1.000	1.288 (0.568)	2.308 (6.929)	1.313 (0.375)	0.929	1.000
**L-Proline**	186.226 (56.889)	188.215 (49.229)	202.916 (43.959)	0.152	1.000	185.135 (55.458)	188.792 (48.981)	197.152 (51.387)	0.426	1.000
L-Arginine *	71.748 (14.970)	69.180 (18.837)	67.686 (13.335)	0.693	1.000	69.609 (17.453)	69.263 (16.657)	66.761 (15.467)	0.798	1.000
**L-Ornithine**	76.410 (23.151)	82.478 (30.591)	97.123 (72.984)	0.411	1.000	73.665 (18.991)	79.683 (24.979)	101.239 (76.291)	0.334	1.000
**L-Lysine**	152.499 (27.927) *	168.823 (31.944)	174.336 (23.859) *	0.025	0.975	155.491 (24.986)	169.067 (34.639)	172.438 (21.154)	0.059	1.000
**L-Valine**	244.565 (45.337) # *	290.103 (53.620) #	291.946 (45.378) *	7.5 × 10^−4^	**0.029**	263.556 (43.649)	280.199 (61.459)	291.348 (42.990)	0.103	1.000
L-Methionine *	28.276 (6.731)	29.574 (7.626)	30.997 (6.562)	0.423	1.000	28.310 (6.625)	29.393 (7.302)	30.841 (7.313)	0.47	1.000
**L-Tyrosine**	69.666 (10.675) # *	78.239 (16.443) #	85.033 (15.838) *	0.001	**0.039**	70.552 (11.647) *	78.122 (17.263)	82.296 (15.671) *	0.03	1.000
**L-Isoleucine**	60.976 (12.066) # *	74.338 (25.326) #	77.534 (16.999) *	8.3 × 10^−4^	**0.032**	66.075 (11.634)	73.088 (27.006)	74.409 (16.780)	0.3	1.000
**L-Leucine**	135.994 (21.617) # *	156.836 (34.849) #	158.108 (20.361) *	0.003	0.117	146.626 (21.119)	151.090 (37.617)	156.774 (19.545)	0.2	1.000
**L-Phenylalanine**	58.192 (6.279) # *	63.528 (8.868) #	65.826 (9.936) *	0.006	0.234	60.222 (7.204)	62.049 (8.665)	66.469 (10.402)	0.117	1.000
**L-Tryptophan**	58.092 (11.694)	63.356 (11.024)	65.707 (12.084)	0.081	1.000	61.585 (9.455)	61.098 (11.580)	65.605 (12.587)	0.2	1.000

Note: * normally distributed variable. *p*-Value was obtained using Kruskal–Wallis with Dunn’s post hoc test for non-normally distributed variables or ANOVA with Tukey’s post hoc test for non-normally distributed variables.; # Differences between low and medium, * differences between low and high, ** differences between medium and high. *p*-Value < 0.05 for multiple comparisons using Benjamini–Hochberg method.; ^c^ Bonferroni correction was used to correct raw *p*-Values (multiplied by 39) for multiple testing, bold *p*-Values show statistical significance. Essential AAs: arginine + histidine + isoleucine + leucine + lysine + methionine + phenylalanine + threonine + tryptophan + valine, nonessential AAs: alanine + asparagine + aspartic acid + cysteine + glutamic acid + glutamine + glycine + proline + serine + tyrosine, GSG index: glutamate/(serine + glycine), BCAAs: valine + leucine + isoleucine, AAAs: tyrosine + phenylalanine.

**Table 3:** The statistically significant associations of AA concentrations with log-PDFF and log-cT1.

Amino Acids (AAs)	Model 1	Model 2	Model 3	Model 4	Model 5
	Exp(Beta) (Corrected *p*-Value ^c^)	Exp(Beta) (Corrected *p*-Value ^c^)	Exp(Beta) (Corrected *p*-Value ^c^)	Exp(Beta) (Corrected *p*-Value ^c^)	Exp(Beta) (Corrected *p*-Value ^c^)
	**Log-PDFF (%)**				
**Essential AAs**	1.002 (0.037)	*NS*	*NS*	*NS*	*NS*
**GSG index**	1.032 (0.019)	*NS*	*NS*	*NS*	*NS*
**BCAAs**	1.003 (0.014)	*NS*	*NS*	1.003 (0.037)	1.003 (0.040)
**AAAs**	1.013 (0.001)	1.012 (0.008)	1.013 (0.006)	1.012 (0.043)	1.012 (0.036)
**L-Glutamic Acid**	1.015 (0.006)	1.014 (0.019)	1.014 (0.025)	1.015 (0.026)	1.016 (0.031)
**L-Valine**	1.005 (0.005)	1.005 (0.018)	1.005 (0.024)	1.005 (0.009)	1.005 (0.010)
**L-Tyrosine**	1.018 (0.003)	1.016 (0.014)	1.017 (0.010)	*NS*	*NS*
**L-Phenylalanine**	1.029 (0.014)	*NS*	*NS*	*NS*	*NS*
	**Log-cT1 (ms)**				
**AAAs**	*NS*	*NS*	1.001 (0.038)	*NS*	*NS*
**L-Threonine**	*NS*	1.001 (0.041)	1.001 (0.008)	1.001 (0.001)	1.001 (0.001)
**L-Glutamic Acid**	1.002 (0.018)	1.002 (0.014)	1.002 (0.043)	1.002 (0.043)	1.002 (0.031)

The PDFF (%) and cT1 (ms) were log-transformed due to the skewness of the distribution. Essential AAs: arginine + histidine + isoleucine + leucine + lysine + methionine + phenylalanine + threonine + tryptophan + valine, GSG index: glutamate/(serine + glycine), BCAAs: valine + leucine + isoleucine, AAAs: tyrosine + phenylalanine. Five adjustment sets were evaluated: Model 1—unadjusted; Model 2—age + sex; Model 3—age + sex + BMI; Model 4—age + sex + BMI + center of the study + smoking + PAL; Model 5—age + sex + BMI + center of the study + smoking + PAL + nutrient intake of the specific AA. ^c^ Bonferroni correction was used to correct raw *p*-Values (multiplied by 39) for multiple testing. In all tests, a *p* value of <0.05 was considered significant. NS: not significant.

## Data Availability

The data described in this study are available from the corresponding author upon request. Due to privacy/ethical constraints on the data submitted by volunteers, the data is not publicly available.

## Supplementary Materials

The following supporting information can be downloaded at: https://www.mdpi.com/article/10.3390/metabo13080959/s1, Table S1: Correlation analysis between amino acid concentrations and MRI parameters, inflammation and oxidative stress biomarkers, biochemical parameters and anthropometrics; Table S2: The associations of AA concentrations with log-PDFF; Table S3: The associations of AA concentrations with log-cT1.
