# Supplementary material for: Plasma Amino Acids in NAFLD Patients with Obesity Are Associated with Steatosis and Fibrosis: Results from the MAST4HEALTH Study
Source: Metabolites. 2023 Aug 18;13(8):959. doi: 10.3390/metabo13080959 (PMC10456787; doi:10.3390/metabo13080959)
Supplement: Supplementary file 1 [file metabolites-13-00959-s001.zip › TableS1.pdf]

Table S1: Correlation analysis between Amino Acid Concentrations and MRI parameters, inflammation and oxidative stress biomarkers, biochemical parameters and anthropometrics. *Pearson's correlation coefficient for normally distributed variables and Spearman's rank correlation for non-normally distributed variables. Note: *normally distributed variable. Correlation coefficient values are given. Yellow color indicates a statistically significant result of correlation analysis. * P ≤ 0.05, ** P ≤ 0.01, *** P ≤ 0.001.*

	MRI parameters			
**Amino Acids (AAs)**	cT1 (ms)	LIF	PDFF (%)	iron ROI pooled median (mg/g)
**Essential AAs**	0.28	0.29	0.38	0.27
**Nonessential AAs**	0.16	0.10	0.13	0.11
**GSG index (glutamate / (serine + glycine))**	0.20	0.27	0.32	0.16
**BCAAs (Valine + Leucine + Isoleucine)**	0.21	0.23	0.38	0.28
**AAAs (Tyrosine + Phenylalanine)**	0.25	0.25	0.44 **	0.32
**L-Alanine***	0.25	0.23	0.16	0.02
**beta-Alanine***	-4.56e-03	0.08	0.10	0.23
**Sarcosine**	0.11	0.18	0.07	-0.06
**Cystine**	0.02	0.05	-0.03	-0.18
**L-Serine**	-0.11	-0.15	-0.05	0.12

**O-Phosphoethanolamine**	0.07	0.12	-0.04	-0.33
**Taurine**	0.10	0.16	-0.02	-0.11
**L-Asparagine**	0.14	0.07	0.09	0.10
**Hydroxy-L-Proline**	0.08	0.03	0.14	0.22
**Glycine**	-0.13	-0.16	-0.27	-0.12
**L-Glutamine***	-8.56e-03	-0.06	0.02	0.13
**Ethanolamine***	9.20e-03	-8.10e-03	0.03	0.19
**L-Aspartic acid**	0.24	0.27	0.25	0.13
**L-Citruline**	-0.04	-0.08	-0.09	0.02
**L-Threonine**	0.27	0.25	0.10	-0.05
**L-Glutamic acid**	0.28	0.35	0.35	0.16
**L-Histidine***	-0.05	0.01	0.02	-8.34e-04
**1-Me-L-Histdine**	0.12	0.14	0.12	0.08
**3-Me-L-Histdine**	0.06	0.05	0.16	0.26
**gamma-Amino-butyric acid GABA**	0.02	0.02	-0.05	-0.11

**D,L-beta-Aminoisobutyric acid**	-0.17	-0.18	-0.29	-2.49e-03
**D,L-alpha-Amino-n-butyric acid**	-0.03	-0.04	0.08	0.09
**L-alpha-Aminoadipic acid**	0.06	0.10	0.29	0.17
**L-Proline**	0.15	0.13	0.19	0.12
**L-Arginine***	-3.45e-03	0.03	-0.01	-0.16
**L-Orthithine**	0.10	0.04	0.12	0.26
**L-Lysine**	0.28	0.23	0.24	0.33
**L-Valine**	0.23	0.27	0.37	0.24
**L-Methionine***	0.12	0.09	0.22	0.11
**L-Tyrosine**	0.26	0.24	0.44 **	0.32
**L-Isoleucine**	0.17	0.16	0.42 *	0.33
**L-Leucine**	0.14	0.14	0.33	0.28
**L-Phenylalanine**	0.23	0.25	0.38	0.27
**L-Tryptophan**	0.12	0.10	0.25	0.27

cT1: included iron-corrected; LIF: liver inflammation fibrosis score; PDFF: proton density fat fraction; iron ROI pooled median: iron region of interest pooled median

Amino Acids (AAs)	Inflammation and oxidative stress biomarkers										
	EGF (pg/mL)	Glut (U/L)	HGB (g/mL)	IL-1a (pg/mL)	IL-1b (pg/mL)	IL-2 (pg/mL)	IL-4 (pg/mL)	IL-6 (pg/mL)	IL-8 (pg/mL)	IL-10 (pg/mL)	IFN (pg/mL)
**Essential AAs**	0.04	0.29	0.32	0.10	-7.71e-04	-0.09	0.04	0.22	0.12	0.03	-0.02
**Nonessential AAs**	0.05	0.24	0.11	0.10	-0.05	0.04	-6.42e-03	-0.02	0.20	0.10	4.36e-03
**GSG index (glutamate / (serine + glycine))**	-0.21	0.03	0.13	0.02	-0.02	-0.09	0.09	0.08	0.07	-0.05	-0.24
**BCAAs (Valine + Leucine + Isoleucine)**	-0.05	0.35	0.33	0.07	-0.02	-0.13	0.05	0.24	0.09	0.05	0.01
**AAAs (Tyrosine + Phenylalanine)**	0.15	0.19	0.22	0.08	0.14	-0.03	0.19	0.19	0.16	0.16	-0.06
**L-Alanine***	5.76e-03	0.07	-0.06	0.10	0.16	6.80e-05	0.09	-0.08	0.06	0.19	0.18

**beta-Alanine***	-0.19	0.14	0.14	0.02	8.12e-03	-0.21	-0.08	0.12	-0.04	0.05	0.03
**Sarcosine**	-0.15	0.06	0.14	1.86e-04	-0.17	-0.13	0.03	0.28	0.11	0.10	-0.12
**Cystine**	0.04	0.14	0.07	0.07	-0.14	0.09	0.04	-0.05	-0.11	-0.19	-0.13
**L-Serine**	0.15	0.06	0.05	-0.02	-0.10	0.04	-0.07	0.06	0.06	0.17	0.02
**O-Phosphoethanolamine**	0.03	-0.05	0.05	-0.14	-0.12	-0.10	-0.09	-3.95e-03	-0.16	-0.07	-0.07
**Taurine**	-0.05	0.18	0.11	-0.09	-0.09	-1.67e-03	0.04	0.10	-0.07	-0.19	-0.37
**L-Asparagine**	0.28	0.04	0.03	0.11	0.11	0.10	-0.08	0.02	0.12	0.17	0.09
**Hydroxy-L-Proline**	0.07	0.03	0.03	-0.10	-0.10	-0.19	-0.03	0.28	-0.06	-0.01	1.99e-03
**Glycine**	0.15	0.02	0.03	0.03	-0.02	0.10	-0.07	-0.10	0.07	0.21	0.09
**L-Glutamine***	-0.02	0.25	0.17	0.09	-0.05	0.05	-0.07	-0.02	0.08	-0.07	3.06e-03
**Ethanolamine***	0.15	0.10	0.41 *	-0.12	0.05	-1.86e-03	-0.04	0.06	-0.05	0.10	-0.02
**L-Aspartic acid**	-0.09	0.05	0.16	-0.15	0.02	-0.17	-0.05	0.21	0.03	0.03	-0.24
**L-Citruline**	0.21	0.09	0.03	-0.15	-0.08	-0.09	-0.27	0.15	0.06	0.04	-0.06

**L-Threonine**	0.06	0.18	0.09	3.30e-03	-0.15	-0.09	-0.05	0.07	0.05	-0.18	-0.24
**L-Glutamic acid**	-0.21	0.03	0.22	0.03	-0.06	-0.10	0.08	0.16	0.17	0.05	-0.22
**L-Histidine***	0.04	0.16	-8.10e-03	0.09	0.06	-0.03	-0.06	0.04	-0.08	-0.03	-0.03
**1-Me-L-Histdine**	-6.92e-03	0.07	0.18	0.10	-0.05	-0.01	0.03	0.10	5.98e-03	0.07	0.04
**3-Me-L-Histdine**	-0.07	0.13	0.22	-0.01	-0.12	-0.15	-0.09	0.20	-0.09	-0.11	0.06
**gamma-Amino-butyric acid GABA**	0.01	-0.17	0.09	-0.11	-0.02	-0.08	-0.03	0.02	-0.01	-0.09	-0.05
**D,L-beta-Aminoisobutyric acid**	0.10	0.11	0.16	-0.14	-0.18	-0.19	-0.08	0.11	-0.05	0.07	-0.10
**D,L-alpha-Amino-n-butyric acid**	-0.05	-0.10	0.13	-0.07	-0.09	0.08	-0.12	0.03	-0.05	-0.04	-0.04
**L-alpha-Aminoadipic acid**	-0.09	0.21	0.21	3.37e-03	0.11	-0.06	0.09	0.19	0.07	-0.04	-0.08
**L-Proline**	-0.03	0.30	0.08	0.02	-0.07	-0.09	0.09	0.06	0.15	0.05	-0.02
**L-Arginine***	-0.20	0.18	-0.11	-7.38e-03	-0.04	-0.07	-0.10	8.78e-03	0.05	-0.12	-0.10

**L-Orthithine**	0.35 (0.278)	-0.04	0.11	7.68e-03	0.05	-0.11	-0.01	0.29	9.38e-03	0.18	0.05
**L-Lysine**	4.14e-03	0.02	0.14	0.14	0.04	3.15e-03	0.12	0.21	0.12	0.13	0.08
**L-Valine**	-0.10	0.36	0.29	0.03	-0.05	-0.16	0.04	0.22	0.06	0.03	0.02
**L-Methionine***	0.23	0.15	0.09	0.01	0.09	-0.05	2.42e-03	0.20	-0.09	0.06	-0.11
**L-Tyrosine**	0.09	0.19	0.13	0.03	0.13	-0.01	0.17	0.19	0.18	0.13	-0.10
**L-Isoleucine**	0.02	0.19	0.28	0.03	-2.07e-03	-0.10	0.05	0.26	0.11	0.02	0.02
**L-Leucine**	0.05	0.29	0.34	0.12	0.04	-0.04	-8.70e-03	0.24	0.07	0.05	0.03
**L-Phenylalanine**	0.19	0.17	0.30	0.15	0.13	-0.05	0.17	0.16	0.08	0.17	0.07
**L-Tryptophan**	0.18	0.22	0.28	-0.02	0.06	0.06	0.14	0.02	0.07	0.14	-0.05

EGF: Epidermal Growth Factor levels; Mean (pg/mL), Glut: Glutathione acivity; Mean (U/L), HGB: Hemoglobin levels; (g/mL), IL-1a: Interleukin-1a levels; Mean (pg/mL), IL-1b: Interleukin-1b levels; Mean (pg/mL), IL-2: Interleukin-2 levels; Mean (pg/mL), IL-4: Interleukin-4 levels; Mean (pg/mL), IL-6: Interleukin-6 levels; Mean (pg/mL), IL-8: Interleukin-8 levels; Mean (pg/mL), IL-10: Interleukin-10 levels; Mean (pg/mL), IFN: Interferon-γ levels; Mean (pg/mL)

Amino Acids (AAs)	Inflammation and oxidative stress biomarkers					
	MCP (pg/mL)	SOD (U/mL)	SOD per HGB (SOD U/mL)(g HGB/mL)	TAS (mmol/L)	TNF (pg/mL)	VEGF (pg/mL)
**Essential AAs**	0.15	0.07	-0.05	0.27	0.16	-0.05
**Nonessential AAs**	-0.01	0.05	-2.38e-04	0.07	0.22	-0.08
**GSG index (glutamate / (serine + glycine))**	0.08	-0.12	-0.15	0.08	-0.16	-0.19
**BCAAs (Valine + Leucine + Isoleucine)**	0.13	0.15	8.50e-03	0.29	0.12	-0.04
**AAAs (Tyrosine + Phenylalanine)**	0.28	0.08	0.02	0.36	0.10	-0.08
**L-Alanine***	0.16	0.10	0.19	0.22	0.08	-0.06
**beta-Alanine***	0.12	-0.07	-0.11	0.19	9.03e-03	-0.08
**Sarcosine**	0.08	-0.13	-0.16	0.09	-0.04	-0.03
**Cystine**	-0.20	-0.20	-0.22	-0.31	0.24	-0.01
**L-Serine**	-0.04	-0.06	-0.11	-0.04	0.16	0.02
**O-Phosphoethanolamine**	0.15	-0.08	-0.08	-0.18	-0.04	0.10
**Taurine**	0.12	-0.16	-0.23	-0.02	0.09	-0.03

**L-Asparagine**	0.08	-0.07	-0.07	0.09	0.21	0.11
**Hydroxy-L-Proline**	8.42e-03	0.25	0.19	0.38	-0.06	0.06
**Glycine**	-0.11	0.08	0.08	-0.06	0.22	0.06
**L-Glutamine***	-0.10	0.09	0.02	0.07	0.11	-0.07
**Ethanolamine***	0.25	0.23	0.09	0.43 **	-0.03	0.02
**L-Aspartic acid**	0.24	-0.13	-0.18	0.26	-0.09	-0.10
**L-Citruline**	-0.01	0.14	0.10	0.14	-9.17e-04	-0.02
**L-Threonine**	0.02	-0.07	-0.10	-0.04	0.06	0.12
**L-Glutamic acid**	0.02	-0.12	-0.19	0.11	-0.07	-0.18
**L-Histidine***	0.08	-0.15	-0.10	0.03	0.07	-0.23
**1-Me-L-Histdine**	0.04	0.26	0.17	0.24	-0.01	0.12
**3-Me-L-Histdine**	0.13	0.25	0.14	0.37	0.10	-0.12
**gamma-Amino-butyric acid GABA**	0.06	-0.19	-0.19	6.52e-03	-0.07	-0.02
**D,L-beta-Aminoisobutyric acid**	-0.08	0.03	-0.02	-0.01	0.11	0.01
**D,L-alpha-Amino-n-butyric acid**	0.04	0.16	0.12	0.23	-0.08	5.73e-03
**L-alpha-Aminoadipic acid**	0.19	0.07	0.04	0.21	-0.12	9.54e-03
**L-Proline**	-0.09	0.05	0.04	0.06	0.14	-0.10
**L-Arginine***	-0.06	-0.03	0.03	-0.15	-0.06	-0.10

**L-Orthithine**	0.26	0.21	0.16	0.44 **	0.15	0.08
**L-Lysine**	0.24	-7.24e-03	-0.04	0.26	0.17	-0.05
**L-Valine**	0.17	0.11	-0.02	0.24	0.12	-0.06
**L-Methionine***	0.13	0.22	0.18	0.31	0.06	-0.01
**L-Tyrosine**	0.26	0.09	0.05	0.35	0.03	-0.05
**L-Isoleucine**	0.06	0.12	0.02	0.33	-1.09e-03	0.02
**L-Leucine**	0.13	0.22	0.07	0.35	0.16	-0.04
**L-Phenylalanine**	0.28	0.06	-0.03	0.32	0.25	-0.13
**L-Tryptophan**	0.21	0.14	0.01	0.34	0.07	-0.12

MCP: Monocyte Chemoattractant Protein1; Mean (pg/mL), SOD: Superoxide dismutase acivity; Mean (U/mL), SOD per HGB: Superoxide dismutase acivity per gram HGB; Mean (SOD U/mL)(g HGB/mL), TAS: Total Antioxidant Status; Mean (mmol/L), TNF: Tumour Necrosis Factor; Mean (pg/mL), VEGF: Vascular Endothelial Growth Factor; Mean (pg/mL)

	**Biochemical parameters**						
**Amino Acids (AAs)**	ALT (IU/L)	AST (IU/L)	AST/ALT ratio	Total cholesterol (mg/dL)	LDL (mg/dL)	HDL (mg/dL)	Triglycerides (mg/dI)
**Essential AAs**	0.33	0.23	-0.28	-0.11	-0.08	-0.23	0.19
**Nonessential** AAs	0.22	0.18	-0.20	-0.03	-6.69e-03	-0.04	-0.05

**GSG index (glutamate / (serine + glycine))**	0.14	0.07	-0.12	-0.18	-0.21	-0.19	0.30
**BCAAs (Valine + Leucine + Isoleucine)**	0.30	0.13	-0.36	-0.05	-0.08	-0.23	0.30
**AAAs (Tyrosine + Phenylalanine)**	0.43 *	0.32	-0.33	-0.16	-0.16	-0.31	0.24
**L-Alanine***	0.13	0.14	-0.08	-0.03	-0.06	-0.16	0.21
**beta-Alanine***	0.08	7.95e-03	-0.15	0.04	0.03	0.02	-0.01
**Sarcosine**	0.03	-0.01	-0.10	-0.10	-0.06	-0.01	-0.06
**Cystine**	3.19e-03	-0.01	-0.04	-0.13	-0.17	0.15	-0.02
**L-Serine**	0.09	0.08	-0.08	0.06	0.11	0.07	-0.21
**O-Phosphoethanolamine**	3.27e-03	-0.03	-0.06	-0.04	-0.10	0.14	3.26e-03
**Taurine**	-0.06	-0.03	0.13	0.08	0.07	0.07	-0.09

**L-Asparagine**	0.15	0.21	-0.02	-0.05	-0.05	0.01	-0.10
**Hydroxy-L-Proline**	0.22	0.18	-0.18	0.03	0.08	-0.16	0.11
**Glycine**	-3.90e-03	-0.02	-7.24e-03	0.06	0.08	0.09	-0.19
**L-Glutamine***	0.15	0.09	-0.15	-0.06	-0.03	0.10	-0.21
**Ethanolamine***	0.25	0.26	-0.12	0.09	0.12	-0.26	0.15
**L-Aspartic acid**	0.06	0.13	0.04	-0.05	-0.05	-0.23	0.20
**L-Citruline**	0.06	8.41e-03	-0.04	0.04	0.02	0.10	-0.03
**L-Threonine**	0.14	0.15	-0.05	-0.09	-0.04	0.14	-0.19
**L-Glutamic acid**	0.22	0.14	-0.13	-0.17	-0.19	-0.22	0.30
**L-Histidine***	3.27e-03	-0.08	-0.03	-0.07	-0.06	-0.02	-4.17e-03
**1-Me-L-Histdine**	0.11	0.12	0.03	0.04	0.07	-0.10	0.08
**3-Me-L-Histdine**	0.21	0.25	-0.09	0.17	0.14	-9.33e-03	0.19

**gamma-Amino-butyric acid GABA**	-0.09	0.02	0.14	-0.02	-0.02	-0.09	-0.03
**D,L-beta-Aminoisobutyric acid**	0.04	-0.07	-0.10	0.05	0.09	-0.05	2.63e-03
**D,L-alpha-Amino-n-butyric acid**	-0.04	-0.04	5.81e-03	0.08	0.13	-4.66e-03	-0.03
**L-alpha-Aminoadipic acid**	0.11	0.06	-0.12	-0.02	-0.04	-0.19	0.26
**L-Proline**	0.21	0.17	-0.21	-0.05	-0.04	-0.12	0.08
**L-Arginine***	0.01	5.48e-03	0.02	-0.03	-0.01	0.05	-0.08
**L-Orthithine**	0.25	0.19	-0.14	0.04	0.08	-0.26	0.15
**L-Lysine**	0.19	0.26	-8.69e-03	-0.01	0.08	-0.22	0.10
**L-Valine**	0.23	0.08	-0.30	5.79e-03	-0.02	-0.18	0.28
**L-Methionine***	0.32 (0.249)	0.24	-0.20	-0.10	-0.07	-0.09	4.68e-03

**L-Tyrosine**	0.39	0.34	-0.24	-0.12	-0.12	-0.25	0.21
**L-Isoleucine**	0.32	0.20	-0.32	-0.11	-0.11	-0.28	0.24
**L-Leucine**	0.34	0.17	-0.35	-0.07	-0.07	-0.25	0.28
**L-Phenylalanine**	0.44 **	0.27	-0.39	-0.17	-0.17	-0.33	0.24
**L-Tryptophan**	0.42 *	0.30	-0.33	-0.19	-0.17	-0.26	0.12

ALT: alanine aminotransferase (IU/L), AST: aspartate aminotransferase (IU/L), LDL: Low-density lipoprotein (LDL) cholesterol concentration (mg/dL), HDL: High-density lipoprotein (HDL) cholesterol concentration (mg/dL)

	**Biochemical parameters**					
**Amino Acids (AAs)**	Glucose (mg/dL)	120 min-OGTT Glucose (mg/dL)	Insulin (µU/mL)	HOMA-IR	γ-gt (U/L)	NFS
**Essential AAs**	0.12	0.04	**0.42 ***	**0.47 ****	0.16	-0.02
**Nonessential AAs**	0.12	0.18	0.18	0.22	-0.03	-0.20
**GSG index (glutamate / (serine + glycine))**	-0.01	0.07	0.25	0.24	0.07	-0.04

**BCAAs (Valine + Leucine + Isoleucine)**	0.20	0.04	0.48 ***	0.52 ***	0.15	0.02
**AAAs (Tyrosine + Phenylalanine)**	0.11	0.28	0.46 **	0.47 **	0.10	-0.09
**L-Alanine***	0.03	0.20	0.24	0.23	-0.02	-0.25
**beta-Alanine***	0.14	0.29	0.01	0.01	0.03	-0.24
**Sarcosine**	0.16	0.03	-0.16	-0.05	0.09	-0.09
**Cystine**	0.11	-0.18	0.26	0.29	3.40e-03	0.12
**L-Serine**	0.08	0.15	-0.15	-0.05	0.01	0.11
**O-Phosphoethanolamine**	-0.06	-0.06	-0.05	-0.12	0.20	-0.04
**Taurine**	-0.10	-0.08	-0.01	-0.08	-0.11	-0.05
**L-Asparagine**	0.07	0.08	-0.07	-4.56e-03	0.10	0.07
**Hydroxy-L-Proline**	-0.05	0.02	0.14	0.16	0.20	-0.07
**Glycine**	-0.03	-0.11	-0.15	-0.16	1.91e-03	-0.04
**L-Glutamine***	0.10	0.18	-0.05	1.79e-03	0.02	-0.04
**Ethanolamine***	0.07	0.14	0.06	0.09	-0.05	-0.04

**L-Aspartic acid**	-0.06	0.10	-0.04	4.66e-03	-0.04	-0.12
**L-Citruline**	-0.02	-0.21	0.18	0.12	0.04	0.02
**L-Threonine**	-0.08	-0.04	0.14	0.16	0.02	-0.03
**L-Glutamic acid**	0.04	0.10	0.25	0.25	0.11	-0.04
**L-Histidine***	0.02	0.12	0.05	0.11	-0.16	-0.08
**1-Me-L-Histdine**	-0.33	-0.29	-0.02	-0.10	0.18	-0.13
**3-Me-L-Histdine**	-8.95e-03	-0.06	0.19	0.21	0.17	-0.04
**gamma-Amino-butyric acid GABA**	-0.02	-0.06	-0.19	-0.16	-0.06	-0.13
**D,L-beta-Aminoisobutyric acid**	5.08e-03	-0.01	-0.22	-0.19	0.04	0.03
**D,L-alpha-Amino-n-butyric acid**	0.05	-0.19	-0.18	-0.12	0.08	0.07
**L-alpha-Aminoadipic acid**	0.04	-0.06	0.33	0.33	0.12	-0.08
**L-Proline**	0.09	0.16	0.44 *	0.43 *	0.01	-0.23
**L-Arginine***	-0.03	-0.15	0.22	0.21	-0.07	-0.15
**L-Orthithine**	-0.18	0.11	0.17	0.11	0.07	-0.09

**L-Lysine**	-1.07e-03	0.17	-0.02	0.05	0.21	0.01
**L-Valine**	0.20	0.03	0.44 **	0.48 **	0.12	0.03
**L-Methionine***	-0.08	-0.03	0.38 *	0.36	-9.54e-03	0.05
**L-Tyrosine**	0.09	0.27	0.40	0.43 *	0.08	-0.02
**L-Isoleucine**	0.19	0.09	0.42 *	0.48 **	0.15	-0.10
**L-Leucine**	0.16	0.02	0.45 **	0.47 **	0.16	0.01
**L-Phenylalanine**	0.08	0.20	0.47 **	0.44 *	0.15	-0.15
**L-Tryptophan**	-0.05	0.05	0.26	0.25	0.07	-0.02

120 min-OGTT Glucose: Oral Glucose Tolerance Test after 120 min from intake (mg/dL), HOMA-IR: Homeostatic model assessment Insulin Resistance, γ-gt: Gamma Glutamyl Transpeptidase (U/L), NFS: NAFLD fibrosis score

	**Anthropometrics**				
**Amino Acids (AAs)**	Weight (kg)	BMI (kg/m^2)	Waist circumference (cm)	Hip circumference (cm)	WHR
**Essential AAs**	0.19	-0.03	0.15	0.06	0.16
**Nonessential AAs**	-0.12	-0.08	-0.11	-0.02	-0.11

**GSG index (glutamate / (serine + glycine))**	0.23	0.12	0.23	0.04	0.28
**BCAAs (Valine + Leucine + Isoleucine)**	0.27	0.05	0.23	0.09	0.24
**AAAs (Tyrosine + Phenylalanine)**	0.12	-0.08	0.10	-0.02	0.18
**L-Alanine***	0.04	-0.01	-0.06	-0.07	5.60e-03
**beta-Alanine***	0.02	-0.18	-0.11	-0.20	0.11
**Sarcosine**	0.08	-0.03	0.10	-0.12	0.27
**Cystine**	0.07	0.22	0.32	0.25	0.12
**L-Serine**	-0.13	-0.07	3.31e-03	0.10	-0.17
**O-Phosphoethanolamine**	3.58e-03	6.92e-03	0.12	-0.04	0.20
**Taurine**	0.09	0.17	0.10	0.06	0.01
**L-Asparagine**	-0.12	-0.15	-0.04	-0.06	-0.04
**Hydroxy-L-Proline**	0.16	0.08	0.12	0.01	0.16

**Glycine**	-0.25	-0.14	-0.23	-0.02	-0.24
**L-Glutamine***	-0.08	-0.09	-0.17	-0.06	-0.15
**Ethanolamine***	0.16	-0.19	-0.05	-0.17	0.18
**L-Aspartic acid**	0.12	-3.18e-03	0.09	-0.09	0.23
**L-Citruline**	0.05	-0.07	-0.03	0.05	-0.04
**L-Threonine**	-0.06	-0.06	0.04	0.10	-0.07
**L-Glutamic acid**	0.18	0.09	0.26	0.08	0.27
**L-Histidine***	-0.11	-0.20	-0.18	-0.14	-0.08
**1-Me-L-Histdine**	0.09	-0.06	0.12	-0.06	0.28
**3-Me-L-Histdine**	0.26	-0.03	0.04	-0.02	0.11
**gamma-Amino-butyric acid GABA**	0.05	0.03	0.14	0.06	0.11
**D,L-beta-Aminoisobutyric acid**	0.03	-0.10	-0.04	-0.05	0.07
**D,L-alpha-Amino-n-butyric acid**	0.10	-0.07	-8.20e-03	-0.02	2.37e-03

**L-alpha-Aminoadipic acid**	0.22	-1.37e-03	0.12	0.09	0.09
**L-Proline**	-4.89e-03	-0.04	3.66e-03	0.03	0.02
**L-Arginine***	0.14	0.11	0.10	0.18	-0.08
**L-Orthithine**	-0.03	-0.12	-0.09	-0.16	0.11
**L-Lysine**	-0.03	-0.11	-0.03	-0.07	8.49e-03
**L-Valine**	0.25	0.10	0.21	0.10	0.17
**L-Methionine***	0.03	-0.21	9.17e-03	-0.09	0.14
**L-Tyrosine**	0.05	-0.08	0.05	-0.03	0.11
**L-Isoleucine**	0.22	-0.07	0.19	0.03	0.27
**L-Leucine**	0.27	2.91e-03	0.22	0.05	0.32
**L-Phenylalanine**	0.23	-0.06	0.18	0.01	0.27
**L-Tryptophan**	0.10	-0.18	0.04	-0.06	0.20

BMI: Body Mass Index of the participants (kg/m^2), WHR: Waist to Hip ratio
